# Pathological changes, distribution and detection of *Brucella melitensis* in foetuses of experimentally-infected does

**DOI:** 10.1080/01652176.2020.1867328

**Published:** 2021-01-14

**Authors:** Mazlina Mazlan, Siti Khairani-Bejo, Hazilawati Hamzah, Nurrul Shaqinah Nasruddin, Annas Salleh, Mohd Zamri-Saad

**Affiliations:** a Department of Veterinary Pathology and Microbiology, Universiti Putra Malaysia, Serdang, Malaysia; b Faculty of Dentistry, Universiti Kebangsaan Malaysia, Kuala Lumpur, Malaysia; c Department of Veterinary Laboratory Diagnosis, Faculty of Veterinary Medicine, Universiti Putra Malaysia, Serdang, Malaysia

**Keywords:** Goat, *Brucella melitensis*, caprine brucellosis, experimental infection, pathology, foetus, vertical transmission

## Abstract

**Background:**

Brucellosis of goats is caused by *Brucella melitensis.* It is a re-emerging zoonotic disease in many countries due to transmission from domestic animals and wildlife such as ibex, deer and wild buffaloes.

**Objective:**

To describe the pathological changes, identification and distribution of *B. melitensis* in foetuses of experimentally infected does.

**Methods:**

Twelve female goats of approximately 90 days pregnant were divided into 4 groups. Group 1 was exposed intra-conjunctival to 100 µL of sterile PBS while goats of Groups 2, 3 and 4 were similarly exposed to 100 µL of an inoculum containing 10^9^ CFU/mL of live *B. melitensis.* Goats of these groups were killed at 15, 30 and 60 days post-inoculation, respectively. Foetal fluid and tissues were collected for bacterial identification (using direct bacterial culture, PCR and immuno-peroxidase staining) and histopathological examination.

**Results:**

Bilateral intra-conjunctival exposure of pregnant does resulted in *in-utero* infection of the foetuses. All full-term foetuses of group 4 were either aborted or stillborn, showing petechiations of the skin or absence of hair coat with subcutaneous oedema. The internal organs showed most severe lesions. Immune-peroxidase staining revealed antigen distribution in all organs that became most extensive in group 4. *Brucella melitensis* was successfully isolated from the stomach content, foetal fluid and various other organs.

**Conclusion:**

Vertical transmission of caprine brucellosis was evident causing mild to moderate lesions in different organs. The samples of choice for isolation and identification of *B. melitensis* are stomach content as well as liver and spleen tissue.

## 1. Introduction

Brucellosis is an important infectious zoonotic disease with worldwide distribution (Seleem et al. 2010). It affects a wide range of animal species as well as humans and is among the most common bacterial zoonotic diseases (Poester et al. 2010; Xavier et al. 2010). The main manifestation of animal brucellosis is reproductive failure, which include abortion, infertility and mastitis (Anka et al. 2014) while human brucellosis often results in a severe chronic debilitating illness (Gorvel 2008; Hartady et al. 2014). The most common species infecting humans is *B. melitensis* and human infections usually follow consumption of raw milk and handling of infected materials.

There are clear epidemiological links between brucellosis in wildlife and brucellosis in livestock and humans (Godfroid et al. 2013). Although *B. melitensis* is rarely reported in wildlife compared to *B. abortus* and *B. suis,* the potential of transmission to livestock and humans remains high with interactions between wildlife and livestock regarded as the most important factors for the transmission. Currently, transmission of *Brucella* spp. from wildlife to humans in the developed countries is linked to the butchering and dressing activities of infected wild or feral pig carcases whereas in developing countries is associated with the infected African buffaloes (Godfroid et al. 2013).

Wildlife reservoirs of infectious diseases such as brucellosis is a major management issue. This is especially so since brucellosis has been eradicated in domestic ruminants from most countries in Europe, and a recent high prevalence of *B. melitensis* infection among Alpine ibex (*Capra ibex*) is of concern (Lambert et al. 2018). A study by Lambert et al. (2018) revealed that 30% of the seropositive wild ibex were at risk of shedding the bacterium at the time of capture, and seropositive females were most at risk to excrete Brucella, corresponding to abortion as reported in the domestic ruminants.

Vaccination with Rev-1 vaccine is an effective method of controlling infection by *B. melitensis* (Alton 1987). However, the recommended vaccination of young replacement animals has failed to control brucellosis in some developed countries and is frequently inapplicable in the developing world. In fact, vaccination of animals, particularly pregnant animals lead to the clinical infection (Blasco 1997). Furthermore, multiplicative and shedding capacity by wild ibex following vaccination with Rev.1 was much higher than goats within 90 days. This is of concern with the transmission of this disease to domestic ruminants and humans (Ponsart et al. 2019).

Studies on brucellosis have been conducted on aborted foetuses that were obtained from natural infection (Yazicioglu and Haziroglu 2000; Ilhan and Yener 2008; Sözmen et al. 2010; Al-Tememy et al. 2013) while experimental infection described the involvement of *B. abortus* (Meador and Deyoe 1986; Meador et al. 1989), not *B. melitensis*. There was no report of abnormal macroscopic foetal development following brucellosis in pregnant animals as similarly reported for Schmallenberg virus infection in cows and goats (Laloy et al. 2017; Konig et al. 2019). However, low birth weights have been reported in humans (Kledmanee et al. 2019). Since in many instances, aborted foetuses are available for diagnosis, understanding the pathological changes, and the distribution and isolation of *B. melitensis* from the various organs of foetus at different times of infection is important to improve diagnosis and minimise transmission to humans. Therefore, this study investigates the progressive development of pathological lesions, the distribution and the isolation of antigen in foetal organs following experimental infection of does with *B. melitensis*.

## 2. Materials and methods

The Institutional Animal Care and Use Committee of Universiti Putra Malaysia had approved the study (Approval: AUP No: R19/2014).

### 2.1. Experimental animals

A total of 12 pregnant does of approximately 90 days pregnant were selected for the study. They were obtained from a farm with no history of brucellosis and were tested negative on the Rose Bengal Plate and the Complement Fixation tests. Both tests were performed according to the OIE and EU requirements where any visible agglutination reaction was considered positive for RBPT while the positive threshold for CFT was 20 ICFTU/mL. Upon arrival, the animals were divided into 4 groups with 3 does per group and each group was kept in separate pen within a house with slated flooring. The pens were well ventilated with room temperature not exceeding 27 °C. They were fed cut grass at 2 kg/goat/day and supplemented feed at 500 g/goat/day. Drinking water was available *ad libitum.*

### 2.2. Inoculum

The *B. melitensis* strain used in this study was obtained from a local outbreak of brucellosis in goats in Malaysia (Plumeriastuti and Zamri-Saad 2012). The isolate was stored as stock culture at −80 °C. The stock culture was thawed and later grown onto Brucella agar by incubation at 37 °C for 4 days. The resulted bacterial colonies were harvested into phosphate buffered saline (PBS), pH 7.4 before the bacterial cell concentration was determined using the McFarland Standard. The final inoculum was prepared at a concentration of 10^9^ CFU/mL of live *B. melitensis*.

### 2.3. Experimental design

At the start of the experiment, goats of Group 1 were exposed intra-conjunctiva to 100 μL of sterile PBS in both eyes. Goats of Groups 2, 3 and 4 were similarly exposed to 100 μL of the inoculum prepared earlier (Gee et al. 2004; Kahl-McDonagh et al. 2006). Does of group 2 were killed 15 days post-infection, group 3 at 30 days post-infection while group 4 at 60 days post-infection. Post-mortem examinations were carried out immediately on the foetuses.

### 2.4. Sampling

Whenever possible, 1 mL of the foetal fluid was aspirated from the gravid uterus using sterile syringes during necropsy. The fluid consisted of both the amniotic and allantoic fluid. Similarly, 1 mL of the stomach content of the foetuses was collected aseptically by aspiration into syringes. These samples, along with the brain, lungs, liver, spleen, stomach, and kidneys, as well as the placenta were collected for bacterial culture, and the subsequent identification of *B. melitensis* using PCR. The same organ samples were fixed in 10% buffered formalin for histopathology and immune-peroxidase studies.

### 2.5. Bacterial isolations and detection of Brucella sp

The samples of foetal fluid and stomach content were directly streaked onto the Brucella agar that was pre-added with Brucella Selective Supplement (Oxoid, Hampshire, England). On the other hand, the organ samples were flamed and placed into sterile zipper plastic bags to minimise contamination. Then, sterile PBS (pH 7.4) was added into the zipper bag at tissue to PBS ratio of 1:2 before the samples were then pounded using mortar and pestle. The resultant mixture was used for bacterial culture and extraction of bacterial DNA. About 10 μL of the tissue mixture was cultured onto Brucella agar that was pre-added with Brucella Selective Supplement (Oxoid, Hampshire, England) and incubated at 37 °C with 5% CO_2_ for 10 days. Bacterial colonies that appeared small, rounded, smooth and translucent, glistening and bluish were highly suggestive of *B. melitensis* (OiE 2009) and were confirmed using PCR (Al-Garadia et al. 2011). The forward and reverse primer sequences were P1 (5′-CATGCGCTATGTCTGGTTAC-3′) and P2 (5′-AGTGTTTCGGCTCAGAATAATC’-3′), which amplified the 252 bp fragment. The results were presented as percentage (%) of positive samples over total number of samples.

### 2.6. Histopathology

All organ samples for histopathological examination were embedded in paraffin wax, sectioned at 4 µm and stained with Harris’ Haematoxylin and Eosin (H&E). The slides were viewed under light microscope (Nikon Eclipse 50i, Tokyo, Japan) installed with Nikon imaging software (NIS-Elements D 3.2, Tokyo, Japan). The histological changes were noted and scored as 0: none, 1: mild, 2: moderate and 3: severe. Specific lesions like necrosis, atrophy, haemorrhage, congestion, infiltration of inflammatory cells and hyperplasia were graded based on these criteria; 0: none, 1: 30% affected, 2: 30–60% affected and 3: > more than 60% affected (Xavier et al. 2010). All samples were duplicated and 5 microscopic fields of each slide were randomly selected for lesion scoring. The scores were reported as the average value of each lesion and average value for overall scoring.

### 2.7. Immune-peroxidase staining

For immune-peroxidase (IP) staining, the paraffin-embedded tissues were sectioned at 4 μm, fixed onto coated slides and left overnight on a hot plate at 37 °C. Then, they were deparaffinized by placing the slides in an oven at 56–60 °C for 15 min before being immersed in 2 series of xylene for 5 min each. Next, the sections were rehydrated by submerging the slides in a series of alcohol starting with 100% alcohol for 6 min followed by 70% alcohol for 3 min and lastly, 50% alcohol for 3 min. The slides were then rinsed in running tap water for 30 sec and were placed in PBS for 15 min. The slides were left to air dry before a liquid blocker slide marker pen was used to draw a line along the periphery of the tissue samples. The slides were then submerged in 10 mM sodium citrate buffer, pH 6.0 followed by heat treatment in a carousel microwave oven at the lowest temperature (50 watt) for 10 min to retrieve the antigen. Then, the slides were cooled to room temperature before rinsing in PBS for 10 min. Inactivation of endogenous peroxidase was achieved by dropping 3% hydrogen peroxide (H_2_O_2_) onto the slides followed by incubation at room temperature for 5 min. The slides were then immersed in PBS for 2 min, followed by dropping of 5% BSA in PBS or blocking buffer onto the slides, which were then incubated in a humidified chamber for 15 min at 37 °C. The hyperimmune serum against *B. melitensis,* that was diluted at 1:100 was added onto the slides and were incubated overnight at 4 °C. Then, the slides were rinsed in PBS for 5 min before the secondary antibody, the goat anti-rabbit immunoglobulin (IgG) with horse-radish peroxidase (HRP) (Abnova, Taipei, Taiwan) diluted to 1:500 was added onto the slides and incubated at 37 °C for 1 h. After that, the slides were washed in PBS for 5 min, developed with DAB + Substrate Chromogen System (Dako, Santa Clara, USA) and counterstained with Harris’ haematoxylin. Control slides utilised normal rabbit serum instead of the primary antibody. The prepared IP slides were viewed under light microscope (Nikon Eclipse 50i, Tokyo, Japan) installed with Nikon imaging software (NIS-Elements D 3.2, Tokyo, Japan). The presence and degree of distribution of the antigen demonstrated by the IP staining was graded as 0: none, 1: focal, 2: multifocal and 3: diffuse (Emikpe et al. 2013). All samples were done in duplicate and 5 microscopic fields were randomly selected for lesion scoring. The scores were documented as average value of each distribution and intensity.

### 2.8. Statistical analyses

Data acquired from the histopathological lesion scoring and IP assessment were expressed as mean and standard error (mean ± SE). Statistical analyses on the non-parametric data were carried out using SPSS version 16.0 by using Kruskal-Wallis one-way analysis of variance and followed up by Mann-Whitney U test in which the significance level was defined as p < 0.05. On the other hand, the results obtained from the bacteriological and molecular methods were expressed as the percentage (%) of positive results following confirmation by PCR.

## 3. Results

### 3.1. Clinical signs

Most infected goats of groups 2 and 3 did not show any clinical sign throughout the study period. However, goats of group 4 showed signs including fever, lethargy, loss of appetite, occasional mastitis and endometritis with profuse bloody, necrotic discharge from the vulva together with expulsion of necrotic caruncles, which lasted several days. The milk appeared curdled. A total of 17 foetuses were obtained from all groups; 3 foetuses were from group 1, 5 from group 2, 4 from group 3 and 5 from group 4. All 5 full-term foetuses of group 4 were dead, either aborted or stillborn while all 3 full-term foetuses of group 1 were born alive.

### 3.2. Macroscopic changes

#### 3.2.1. External macroscopic changes

All does in the negative control group 1 delivered healthy kids that showed no gross external lesions associated with brucellosis. However, there were notable external gross lesions related to caprine brucellosis in foetuses from the infected does. All foetuses of group 2 displayed petechial haemorrhages on their bodies. The liver, which was visible through the translucent foetal skin, appeared enlarged and dark red in colour. The placenta exhibited obvious petechial haemorrhages of the placentomes involving approximately 60% of total placentomes.

**Figure 1.**
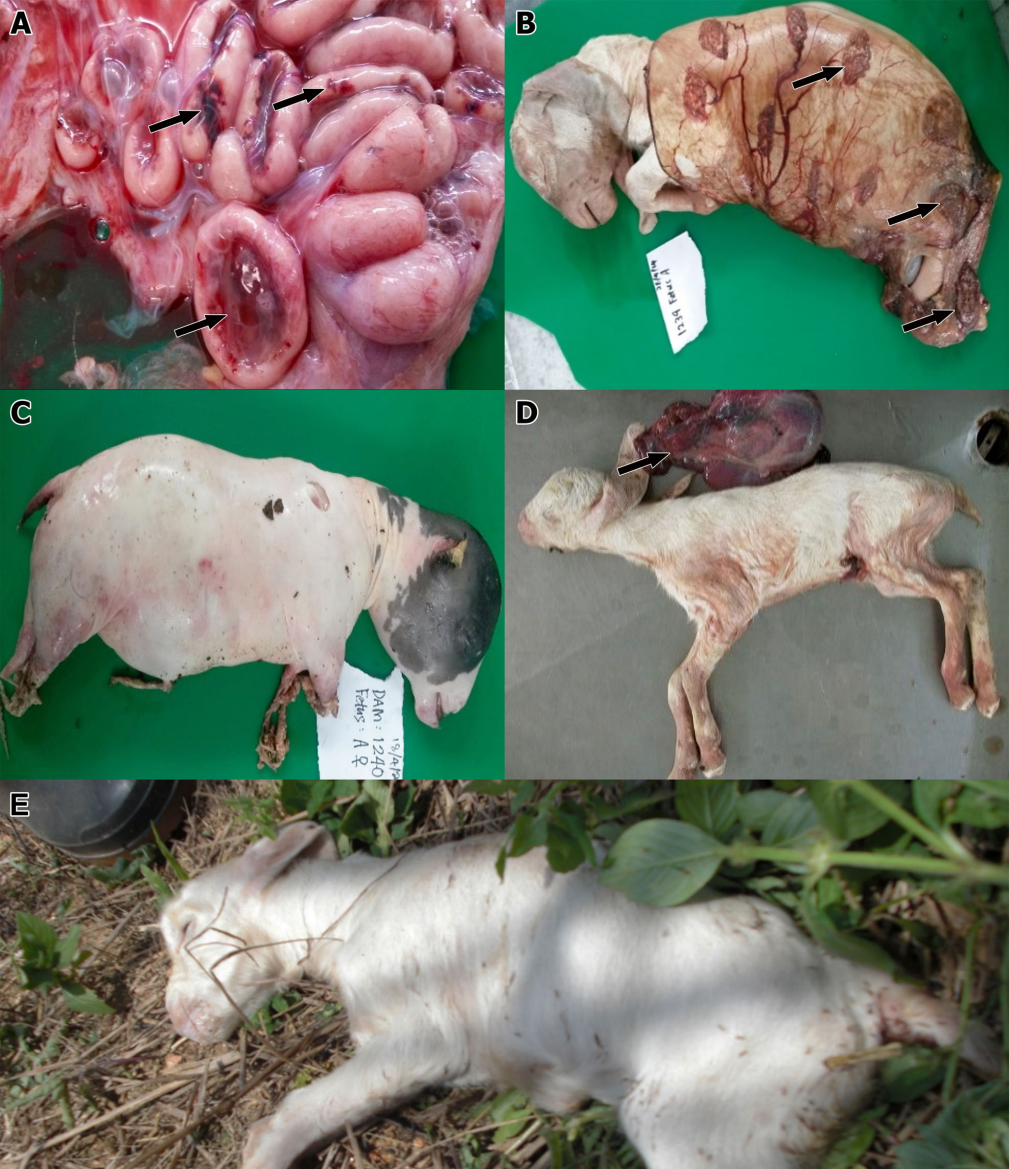
Placentome and foetuses of does experimentally infected with *Brucella melitensis.* (A) Foetal membrane of a foetus of group 3 that was killed after 30 days of infection showing haemorrhagic and necrotic placentome (arrows). (B) A foetus still wrapped in the foetal membrane from a doe of group 4 that was stillborn after 60 days of infection. There was evidence of necrosis of the placentomes, which appear dull (arrows). (C) An aborted foetus from an infected doe of group 4 showing severely oedematous carcase. (D) Full-term but stillborn foetus with normal foetal body but necrotic and reddened placenta (arrow). (E) Full-term normal foetus delivered by a non-infected doe of group 1.

The foetuses of group 3 revealed visible outline of the enlarged liver. The placenta exhibited generalised reddening of the placentomes (Figure 1A). External examination of a full–term but aborted foetus of group 4 revealed a foetus that was still covered by the placenta with severely necrotic, dull and greyish cotyledon (Figure 1B). The same foetus appeared oedematous with subcutaneous accumulation of fluid, particularly at the neck, inguinal and testicular regions. Similarly, another aborted foetus of group 4 showed severe generalised oedema and absence of hair coat (Figure 1C). The remaining full-term but stillborn foetuses of group 4 showed fairly normal foetal body without oedema (Figure 1D) but with severely necrotic and reddened placenta, particularly at the cotyledons.

#### 3.2.2. Visceral macroscopic changes

Generally, foetuses of group 2 showed no remarkable gross changes except for the mildly swollen liver. However, the foetuses of group 3 had congested livers, which were moderately enlarged and lungs with mild ‘cobblestone’ appearance. There was also presence of blood-tinged fluid in the thoracic and abdominal cavities as well as in the intestinal lumen. It was noted that the stillborn foetuses of group 4 exhibited gross lesions such as severe hepatomegaly, lungs with moderate ‘cobblestone’ appearance and accumulation of blood-tinged fluid in the thoracic and abdominal cavities. The intestinal content was watery, brownish red in colour. The aborted foetuses were severely oedematous and necrotic with watery, reddish brown content in the intestines. The lungs showed prominent lobular pattern with copious blood-tinged fluid in the cavities of the thorax and abdomen. The intestines contained normal looking semi-solid brownish coloured faecal material.

### 3.3. Microscopic changes and antigenic distribution

All foetuses of the negative control group 1 exhibited no histological changes related to brucellosis. On the contrary, varying degree of microscopic changes associated with infection by *B. melitensis* were noted in various organs of the foetuses from infected groups 2, 3 and 4. Results of the histopathological lesion scoring are presented in Table 1 whereas the immunohistochemistry assessments are summarised in Table 2.

**Table 1.** Average histopathological lesion score (mean ± SE) of various organs from foetuses of pregnant does experimentally infected by *B. melitensis.*

Organ	Group 1 (n = 3)	Group 2 (n = 5)	Group 3 (n = 4)	Group 4 (n = 5)
Brain	0.0 ± 0.0^a^	0.2 ± 0.2^a^	0.3 ± 0.2^a^	0.9 ± 0.4^a^
Lungs	0.0 ± 0.0^a^	0.2 ± 0.1^a^	0.6 ± 0.3^a^	1.3 ± 0.5^a^
Liver	0.0 ± 0.0^a^	0.9 ± 0.4^b^	1.2 ± 0.5^b^	1.4 ± 0.5^b^
Spleen	0.0 ± 0.0^a^	0.4 ± 0.2^a^	0.6 ± 0.3^b^	1.2 ± 0.5^b^
Stomach	0.0 ± 0.0^a^	0.1 ± 0.1^a^	0.3 ± 0.1^b^	1.2 ± 0.5^c^
Kidneys	0.0 ± 0.0^a^	0.2 ± 0.2^a^	0.6 ± 0.2^b^	1.2 ± 0.4^c^
Placenta	0.0 ± 0.0^a^	0.4 ± 0.1^b^	0.6 ± 0.2^b^	1.4 ± 0.5^b^

Group 1 is the negative control, group 2 was infected for 15 days, group 3 for 30 days and group 4 for 60 days.

^a,b,c^Different superscripts in the same row indicate significant (p < 0.05) differences.

**Table 2.** Average immuno-peroxidase staining score (mean ± SE) in the various organs of foetuses from pregnant does experimentally infected by *B. melitensis.*

Organ	Group 1 (n = 3)	Group 2 (n = 5)	Group 3 (n = 4)	Group 4 (n = 5)
Brain	0.0 ± 0.0^a^	0.0 ± 0.0^a^	0.2 ± 0.2^a^	2.0 ± 0.0^b^
Lungs	0.0 ± 0.0^a^	0.9 ± 0.5^a^	1.3 ± 0.1^b^	2.7 ± 0.3^c^
Liver	0.0 ± 0.0^a^	2.4 ± 0.1^b^	2.7 ± 0.2^b^	2.9 ± 0.1^b^
Spleen	0.0 ± 0.0^a^	0.8 ± 0.1^b^	1.5 ± 0.1^c^	2.7 ± 0.3^d^
Stomach	0.0 ± 0.0^a^	0.3 ± 0.1^b^	1.3 ± 0.1^c^	2.8 ± 0.1^c^
Kidneys	0.0 ± 0.0^a^	0.6 ± 0.1^b^	2.1 ± 0.1^c^	2.5 ± 0.2^c^
Placenta	0.0 ± 0.0^a^	1.5 ± 0.1^b^	1.9 ± 0.1^b^	2.9 ± 0.1^c^

Group 1 is the negative control, group 2 was infected for 15 days, group 3 for 30 days and group 4 for 60 days.

^a,b,c^Different superscripts in the same row indicate significant (p < 0.05) differences.

The foetal brain of group 2 had mild congestion of the blood vessels of the brain. Moreover, immune-peroxidase showed no immunoreaction towards *B. melitensis.* The foetal brain of group 3 exhibited lesions such as moderately congested with presence of inflammatory cells, mostly mononuclear cells. Immune-peroxidase staining revealed multifocal, moderately strong brown deposits. The foetal brain of group 4 showed most severe microscopic changes with meningoencephalitis following infiltration of mononuclear inflammatory cells, severe congestion, mild haemorrhages and necrosis of the neurons with cerebral vacuolation and perivascular oedema. The inflammatory cells showed positive intracytoplasmic immunoreaction. Nevertheless, there was no significant (p > 0.05) differences in the average scoring of lesions in the foetal brain of all groups. However, the distribution of the *Brucella-*specific antigen in foetal brain of group 4 was significantly (p < 0.05) more extensive than other groups.

**Figure 2.**
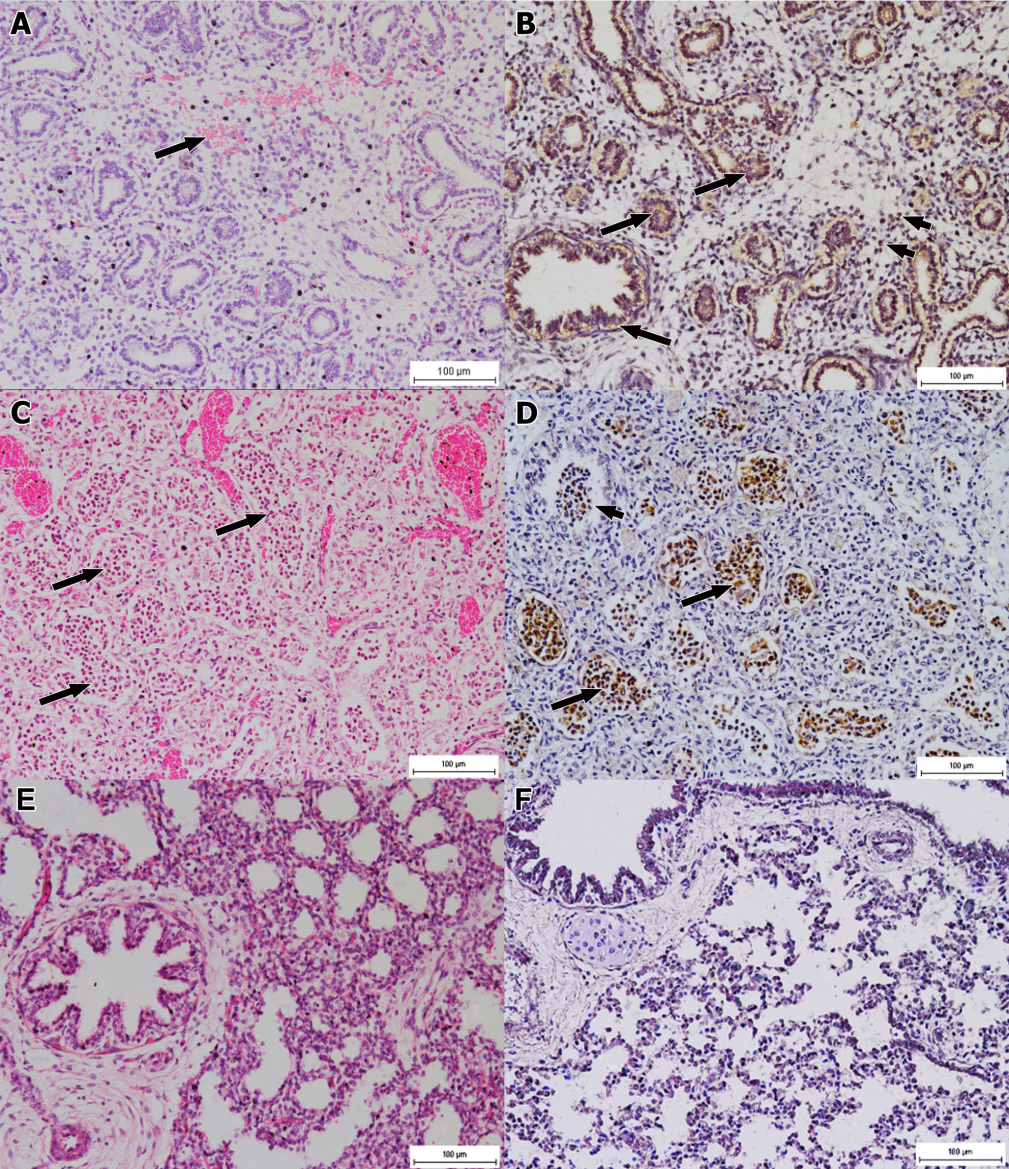
Photomicrograph of lung sections. (A) Mild inflammation and haemorrhage (arrow) in the lungs of a foetus of group 2 after 15 days of infection. HE x200. (B) The lungs section showing distribution of *B. melitensis,* particularly in the epithelium of bronchioles (long arrows) and few macrophages (short arrows). IP x200. (C) Severe and extensive broncho-interstitial pneumonia (arrows) in the lung section of a foetus of group 4 after 60 days of infection. The inflammatory cells are mostly macrophages. HE x200. (D) *Brucella melitensis* is found mainly within the macrophages either in the alveolar space (long arrows) or lumen of bronchioles (short arrows). IP x200. (E) Relatively normal lung section of a foetus of non-infected group 1. HE x200. (F) Lung section of a foetus of non-infected group 1 showing negative immune-peroxidase reaction. IP x200.

There were prominent microscopic changes in the lungs of foetuses of infected does and the severity was parallel with the chronicity of the infection. Foetuses of group 2 showed less remarkable microscopic changes with mild haemorrhages, congestion and infiltrations of inflammatory cells (Figure 2A). Thus, IP staining showed presence of mild golden brown staining in the lung tissues, particularly in the cytoplasm of epithelial cells of the bronchioles and in a few macrophages (Figure 2B). On the other hand, the foetal lungs of group 3 showed moderate interstitial pneumonia and congestion leading to thickening of the alveolar septa. There were mild haemorrhages and necrosis of the epithelial linings of some bronchioles. Immune-peroxidase staining revealed the presence of *Brucella* antigen in the lungs of foetuses of group 3. As expected, foetuses of group 4 exhibited the most severe lesions with severe necrosis and loss of lung architecture while other parts of the lungs showed broncho-interstitial pneumonia with intense infiltration of mononuclear inflammatory cells, necrosis of the epithelial lining of the bronchioles and alveoli, severe congestion and mild haemorrhages (Figure 2C). The immune-peroxidase staining demonstrated the presence of strong brown staining intracellularly in the macrophages, in the alveolar spaces and the lumen of bronchioles (Figure 2D). The degree of inflammatory cell infiltration in the foetal lungs was significantly (p < 0.05) different between all groups but for necrosis and congestion in the lungs of group 4 was significantly (p < 0.05) more severe compared to other groups. Similarly, the distribution of IP staining of the foetal lungs of group 4 was significantly (p < 0.05) more than other groups.

**Figure 3.**
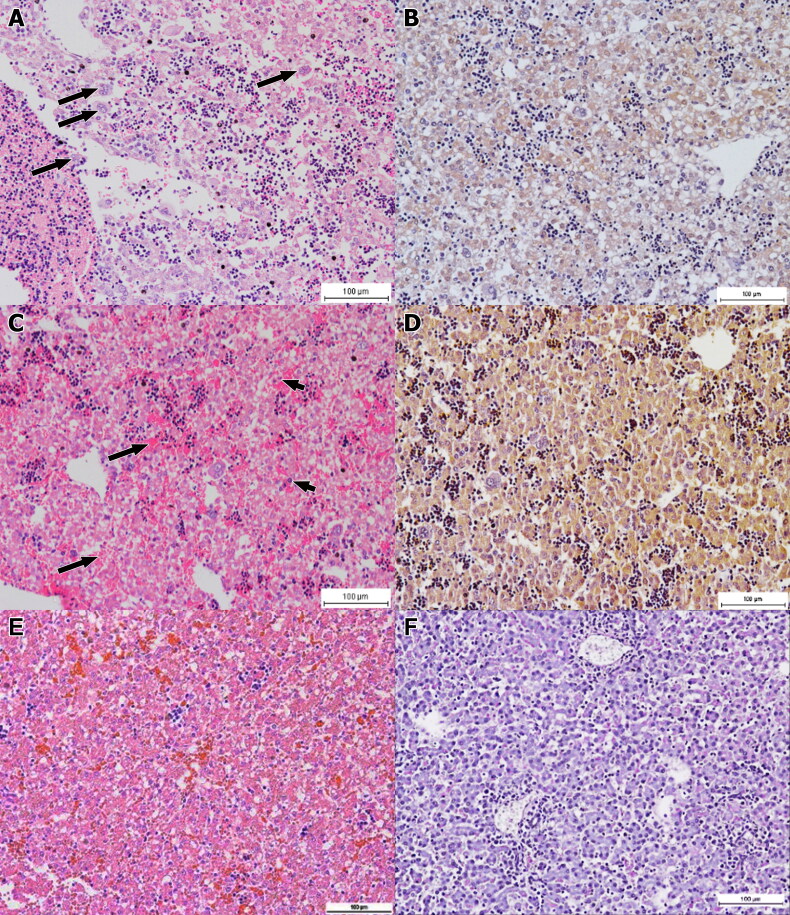
Foetal liver sections. (A) Moderately severe hepatic necrosis with foci of inflammation consisted of macrophages throughout the liver section of a foetus of group 2. Note the presence of EMH with megakaryocytes (arrows). HE x200. (B) Mild distribution of *B. melitensis*in foetal liver after 15 days of infection. IP x200. (C) Foetal liver section from doe of group 3 that was infected for 30 days. Moderate congestion (long arrows) and inflammation (short arrows) with EHM and megakaryocytes. HE x200. (D) Moderately diffuse distribution of *B. melitensis*in the foetal liver of group 3. IP x200. (E) Foetal liver of non-infected group 1 showing relatively normal liver. HE, x200. (F) Negative immuno-peroxidase staining in the foetal liver section of group 1. IP, x200.

Varying degree of hepatitis was observed in the foetal liver of *B. melitensis*-infected does, and group 4 was generally showing most severe lesion. The affected livers showed evidence of extramedullary haematopoiesis (EMH) with presence of clumps of precursor cells and megakaryocytes in the sinusoids. The liver of foetuses of group 2 showed intense EMH activities with severe congestion while some hepatocytes showing karyolysis and vacuolation. There were macrophages and mild haemorrhage in the sinusoids (Figure 3A). Immune-peroxidase staining revealed presence of mild brownish staining that was well distributed in the liver (Figure 3B). Similarly, the foetuses of group 3 had liver with intense EMH and necrosis of hepatocytes, accompanied with severe haemorrhages in the sinusoids (Figure 3C). Immune-peroxidase evaluation revealed strong positive staining, affecting the whole liver but not the megakaryocytes (Figure 3D). The foetuses of group 4 displayed the most severe lesions in the liver in the form of severe necrosis of the liver and immune-peroxidase staining showed intense stain throughout the entire liver. As expected, the degree of necrosis in group 4 was significantly (p < 0.05) more severe than all other groups. The immune-peroxidase staining distribution in the liver of foetuses was significantly (p < 0.05) higher between groups.

There was evidence of inflammation in the foetal spleen of all infected groups 2, 3 and 4. Group 2 showed moderate congestion, splenic hyperplasia due to mild infiltration of mononuclear cells in the red pulp and mild EMH. Immune-peroxidase staining showed presence of multifocal areas of mild light brown deposits, particularly in the cytoplasm of the macrophages. Group 3 displayed more obvious microscopic changes, which included mild necrosis, moderate splenic congestion and moderate hyperplasia of the lymphoid follicles while the red pulp showed influx of macrophages besides mild EMH. These findings were supported by the more diffuse but mildly positive IP reactions in the red pulp, in the cytoplasm of macrophages. On the contrary, the spleen of foetuses of group 4 showed the most pronounced microscopic changes. This was evident from the lesions such as moderate splenic necrosis and congestion, severe hyperplasia of the white pulp with influx of macrophages in the red pulp accompanied by moderate EMH as seen from the presence of many megakaryocytes and clumps of precursor cells. There was intense golden brown positive IP reactions, found intracellularly in macrophages within the red pulp. Statistically, splenic hyperplasia was found to differ significantly (p < 0.05) between all groups whereas the necrosis, congestion and inflammatory cell infiltration in group 4 were significantly (p < 0.05) more severe compared to other groups. However, the immune-distribution of *B. melitensis* were more extensive (p < 0.05) in groups 3 and 4.

**Figure 4.**
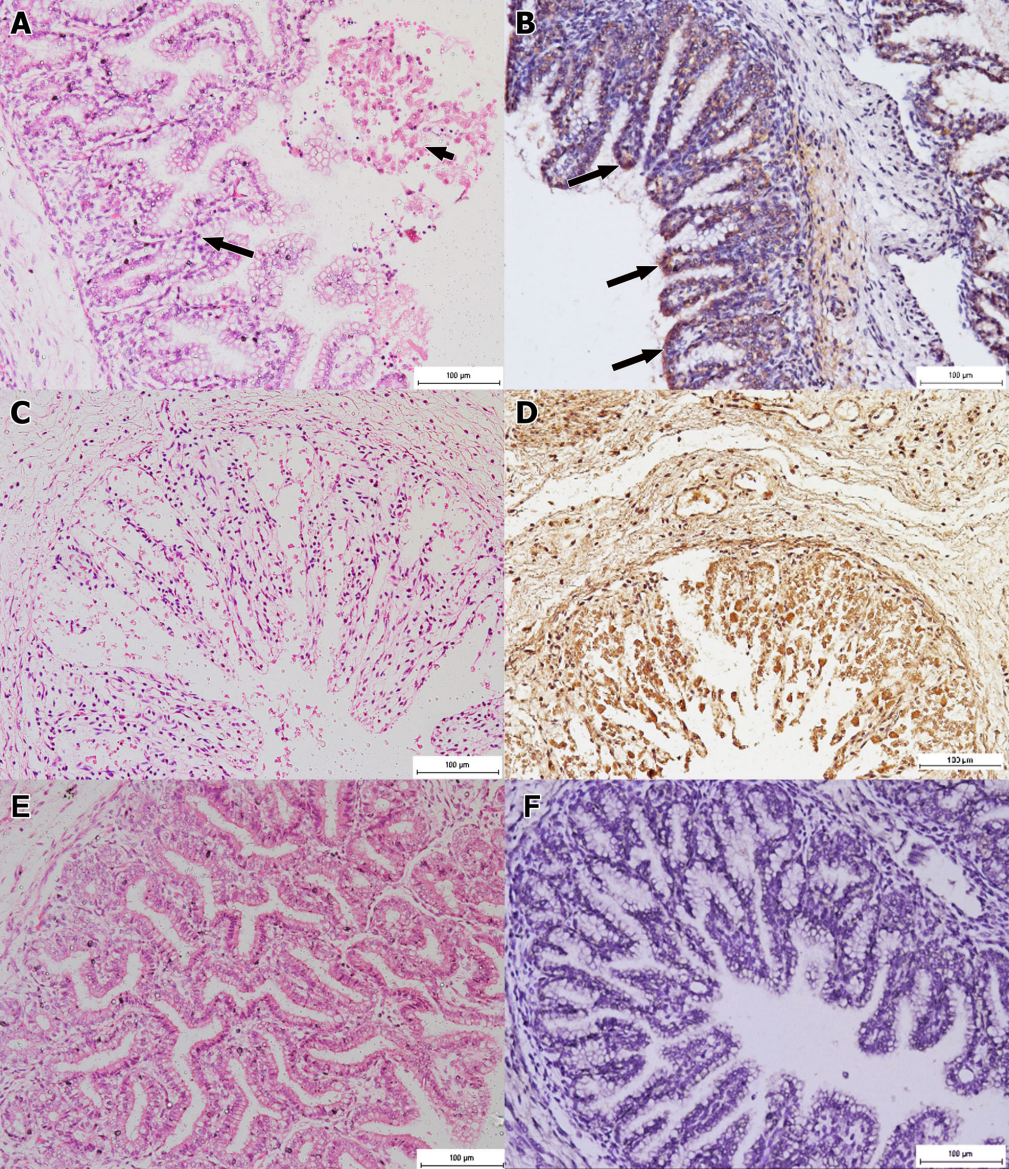
Histology section of foetal stomach of group 3 (A, B) and 4 (C, D). (A) Section of foetal stomach of group 3 that was infected for 30 days showing moderate necrosis and inflammation (long arrows). Note the necrotic remnant in the lumen (short arrows). HE x200. (B) Mild positive immuno-peroxidase staining mostly at the epithelial cells of the stomach (arrows). IP x200. (C) Foetal stomach section of group 4 that was infected for 60 days showing extensive necrosis and inflammation of the stomach. HE x200. (D) Intense and widely distributed immuno-peroxidase staining in the entire stomach section. IP x200. (E) Normal histological feature of the stomach of a foetus of non-infected group 1. HE x200. (F) Negative immune-peroxidase staining of the stomach of a foetus of non-infected group 1. IP x200.

Basically, the stomach of foetuses of group 2 exhibited mild mononuclear inflammatory cell infiltration, particularly in the submucosal layer and the lamina propria. Immune-peroxidase staining revealed pinpoint distribution of brown deposits, involving the stomach epithelium. The foetuses of group 3 showed slight haemorrhage into the lumen, mild congestion and influx of mononuclear cells notable in the submucosa and the lamina propria. There was necrosis of the epithelium with presence of necrotic materials and mononuclear inflammatory cells in the gastric lumen (Figure 4A). The IP assessment revealed strong positive stain that concentrated especially on the epithelial cells of the stomach (Figure 4B). Fascinatingly, foetuses of group 4 developed severe and generalised necrotic gastritis leading to severe haemorrhages into the lumen with moderate congestion and intense mononuclear inflammatory reaction (Figure 4C). The IP staining revealed intense brown staining of the entire stomach tissue (Figure 4D). Lesions such as necrosis and inflammatory reaction of group 4 were significantly (p < 0.05) more severe than groups 1–3. However, IP staining showed significant (p < 0.05) differences in the distribution of the antigen amongst all groups.

The kidneys of foetuses of group 2 developed mild glomerulonephritis by mononuclear cells, renal congestion and haemorrhage. The presence of *B. melitensis* in the kidneys was demonstrated by IP staining as multifocal reactions in the tubular-interstitial tissues and occasionally the glomeruli. The foetuses of group 3 showed mild to moderate glomerulonephritis with moderate renal congestion, mild necrosis and tubular-interstitial haemorrhages. Immune-peroxidase staining was diffuse and moderately strong in both the glomeruli and epithelial cells of the renal tubules. There was severe necrotic glomerulonephritis in the kidneys of foetuses of group 4 apart from the moderate inflammatory cell infiltration, congestion and haemorrhages. Immunoreaction towards *B. melitensis* was diffusely distributed in the kidneys. The renal necrosis and inflammatory reaction, and the distribution and intensity of immune-peroxidase staining in groups 3 and 4 were significant (p < 0.05) more than other groups.

**Figure 5.**
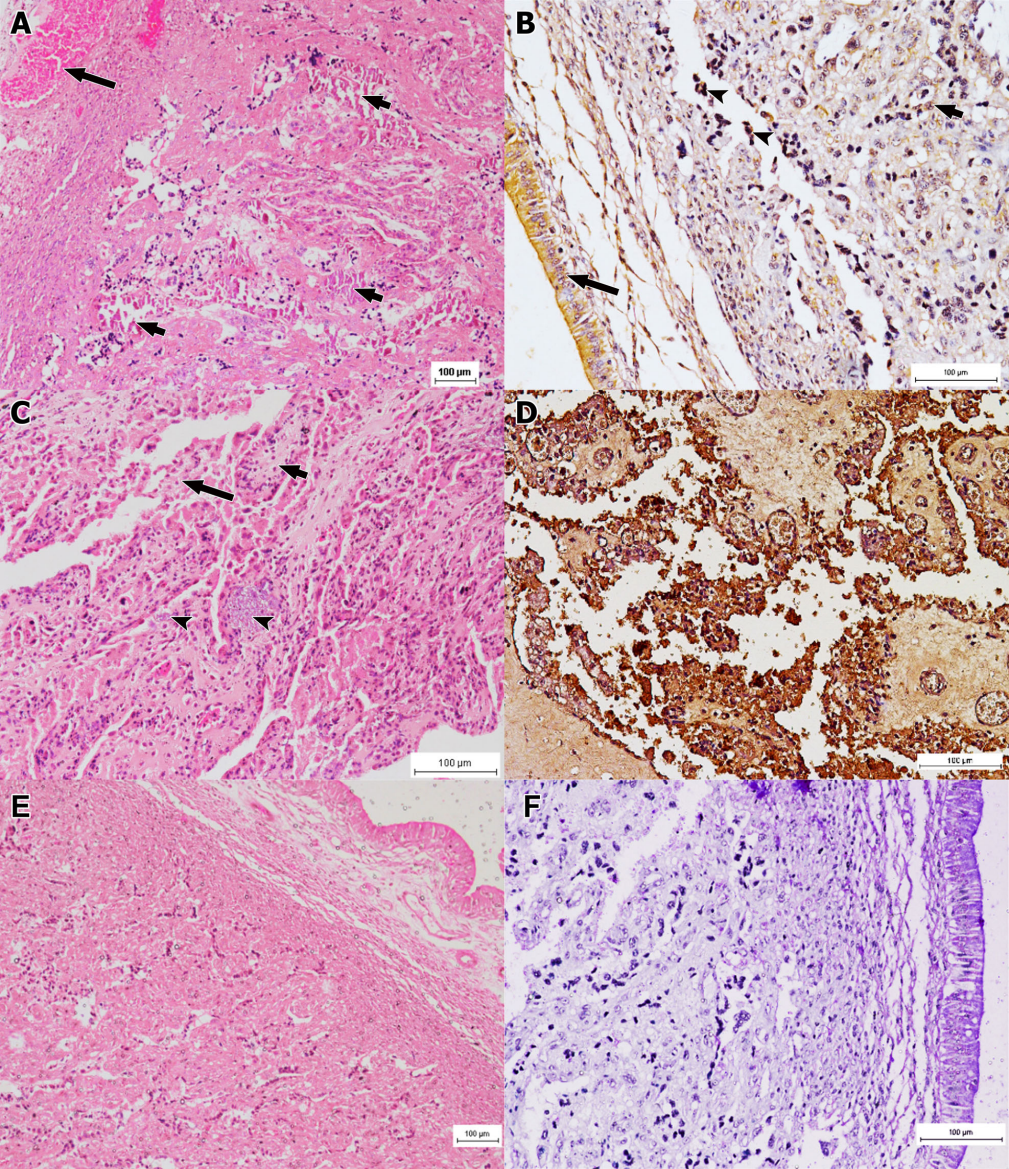
Foetal membrane of doe infected with *B. melitensis.* (A) Placentation of group 3 with severe congestion (long arrows) and accompanied by mild villus necrosis characterised by loss of normal architecture that has been replaced by eosinophilic debris (short arrows) and mild cellular infiltrates. HE, x100. (B) The trophoblasts (long arrows), endometrial glands (short arrow), and the inflammatory cells (arrowheads) showing the light golden brown staining indicating the presence of *Brucella* antigen in the placentomes. IP x200. (C) Severely necrotic (long arrows) and moderately oedematous villi (short arrows) of placenta of group 4 as seen from the loss of normal villus architecture and presence of eosinophilic materials accompanied by severe cellular infiltrate and presence of purplish bacterial colonies (arrowheads). HE, x200. (D) Generally diffuse and intense golden brown staining of the placenta. IP x200. (E) Placenta of a non-infected doe of group 1 that appears normal. HE x200. (F) Placenta of a non-infected doe of group 1 showing negative immune-peroxidase staining. IP. X200.

The placentas of infected does developed remarkable histological lesions of necrotising placentitis, which progress to be more severe with time of infection. The placenta of group 2 showed slightly expanded lamina propria and inter-cotyledonary areas. There was mild immune-peroxidase staining, particularly at the endometrial glands, inter-cotyledonary areas and some of the trophoblasts. The placenta of group 3 displayed severe congestion and haemorrhage accompanied by mild villus necrosis characterised by loss of normal architecture, which had been replaced by eosinophilic debris and mild cellular infiltrates (Figure 5A). The endometrial glands, trophoblasts and cellular infiltrates exhibited the characteristic light golden brown IP staining. Besides that, the placentation displayed moderate expansion of the lamina propria and inter-cotyledonary areas (Figure 5B). Group 4 had the most severe histological lesions. The placenta was severely necrotic and there was moderately oedematous villi as seen from the loss of normal villus architecture and presence of eosinophilic materials accompanied by severe cellular infiltrate and presence of purplish bacterial colonies (Figure 5C). The IP staining revealed diffuse and intense staining in the placenta (Figure 5D). Statistically, the placental necrosis, haemorrhage and congestion were significantly (p < 0.05) severe in group 4. However, the inflammation was significantly (p < 0.05) more severe in groups 3 and 4. The IP staining was significantly (p < 0.05) distributed in the placenta of group 4.

### 3.4. Bacterial culture

None of the stomach content and foetal fluid samples obtained from the control uninfected group 1 and the infected group 2 yielded *B. melitensis*. However, 2 (50%) of the foetuses of group 3 were positive for isolation of *B. melitensis* from both foetal fluid and stomach content while successful isolation of *B. melitensis* was made from the stomach content of all 5 foetuses of group 4.

Table 3 summarises the results for direct organ culture for isolation of *B. melitensis*. None of the foetal organs samples obtained from the control uninfected group 1 yielded *B. melitensis*. Nevertheless, as time progress, greatest number of positive isolations were made from organ samples of foetus of group 2, followed by group 3 and group 4. In group 2, positive isolations were made from the lungs, liver and spleen, group 3 from lungs, liver, spleen, stomach, kidneys and placenta and group 4 from brain, stomach, kidneys, placenta, lungs, liver and spleen. The foetal organs with highest isolation rate were the liver and spleen, followed by the lungs, stomach, kidneys and placenta and the least from the brain (Table 3).

**Table 3.** Percentage (%) of foetus showing positive isolation of *Brucella melitensis* from various organs.

Organ	Group 1 (n = 3)	Group 2 (n = 5)	Group 3 (n = 4)	Group 4 (n = 5)
Brain	0	0	0	60
Lungs	0	40	75	100
Liver	0	60	75	100
Spleen	0	60	75	100
Stomach	0	0	75	60
Kidneys	0	0	50	60
Placenta	0	0	50	60

Group 1 is the negative control, group 2 was infected for 15 days, group 3 for 30 days and group 4 for 60 days.

### 3.5. PCR on foetal organs

Table 4 summarises the percentage of positive bacterial DNA extracted from foetal organs of experimentally infected does. All foetuses of group 1 remained negative while all foetuses of groups 2, 3 and 4 showed positive results. The lowest bacterial DNA detection rate was from organs of foetuses of group 2 followed by group 3 and group 4. Only the foetal brain of group 2 remained negative for brucellosis, other organs showed positive detection in the lungs, stomach and kidneys and all of the liver, spleen and placental samples.

**Table 4.** Percentage (%) of foetus showing positive PCR for *Brucella melitensis* in various organs.

Organ	Group 1 (n = 3)	Group 2 (n = 5)	Group 3 (n = 4)	Group 4 (n = 5)
Brain	0	0	40	100
Lungs	0	60	100	100
Liver	0	100	100	100
Spleen	0	100	100	100
Stomach	0	60	100	100
Kidneys	0	60	60	100
Placenta	0	100	100	100

Group 1 is the negative control, group 2 was infected for 15 days, group 3 for 30 days and group 4 for 60 days.

## 4. Discussion

Following exposure of pregnant does to live *B. melitensis,* all full-term foetuses of group 4 that were chronically infected were either aborted or stillborn. Grossly, the placenta appeared to be severely necrotic. Acute and subacute infected foetuses showed various degree of gross and histopathological changes in the various organs due to the colonisation by *B. melitensis*. These were confirmed by direct bacterial culture, PCR and immuno-peroxidase staining of selected foetal organs. Grossly, affected foetus showed subcutaneous oedema, having blood-stained fluid in the thorax and abdomen that were accompanied by splenomegaly and hepatomegaly as reported earlier (SCAHAW 2001).

Oral is the most common route of infection by *B. melitensis.* However, regardless of the route of infection the organism always end-up in the cervical lymph node with subsequent stimulation of the defence mechanism in the form of gut-associated lymphoid tissue (GALT), producing IgA and activating macrophages. Infection by *Brucella* activates the participation of macrophages, dendritic cells, and CD4(+) and CD8(+) T cells (Dorneles et al. 2015). *Brucella* would be captured by the macrophages and other phagocytic cells but able to survive in the phagocytic cells. The outcome of *Brucella* infection relies on the balance of cytokines produced by Th1 and Th2 cells. The Th1 cytokines, such as the TNF-α and IFN-γ induce the CD8+ CTL-mediated cytotoxicity against *Brucella*-infected macrophage, whereas Th2 cytokine such as the IL-10 activates antibody production by the B lymphocytes, facilitating opsonisation of *Brucella* organism (Choudhary et al. 2019). However, failure of the immune response to prevent the spread of *Brucella melitensis* to the foetus resulted in colonisation in almost all foetal organs (Higgins et al. 2017). The infection usually becomes severe after 2 weeks and would last for a period of 5 to 6 weeks (Lopez-Santiago et al. 2019). Similarly, vaccination with Rev 1 stimulated marked serological response but also resulted in generalised infection 2 weeks post-vaccination that would last for 3 months (Munoz et al. 2008).

In cases of brucellosis, stomach contents of the aborted or stillborn foetuses, are the recommended sample for bacterial culture to diagnose the disease as *B. melitensis* was successfully isolated from the stomach content of all foetuses in this study (OiE 2009; Al-Tememy et al. 2013). However, the stomach contents are of low diagnostic value in acute brucellosis as the successful isolation rate was 0% after 15 day and 33% after 30 days. On the other hand, foetal brain is not recommended for isolation although lesions of meningoencephalitis might develop (Cetín et al. 2004; Sözmen et al. 2010).

The foetal liver has been reported as the organ of choice in diagnosis of brucellosis using aborted foetuses (OiE 2009). There were obvious hepatomegaly and hepatitis with presence of EMH and necrosis of the hepatocytes, similar to the finding reported in naturally occurring aborted foetuses with necrotic hepatitis (Cetín et al. 2004; Al-Tememy et al. 2013) that could be accompanied by EMH (Sözmen et al. 2010). *B. melitensi*s was found mainly in the cytoplasm of inflammatory cells, Kupffer cells and hepatocytes but not in the megakaryocytes (Ilhan and Yener 2008). Furthermore, direct bacterial culture and PCR are reliable in detecting the presence of *B. melitensis* therefore, this study also concluded that foetal liver is the target organ of *B. melitensis*.

Foetal spleen is another target organ for *Brucella* and one of the recommended samples to be cultured to confirm the presence of *B. melitensis* (OiE 2009), which is in agreement with the findings of this study when the foetal spleen was found to be among the first organs to be colonised and confirmed by bacterial culture and PCR as early as day 15 PI. In fact, inflammation of the spleen is reported to be the common sequelae of brucellosis in bovine foetuses (Gorvel 2008). *Brucella melitensis* in this study, was found within the macrophages in the red pulp of the foetal spleen.

Much focus was given towards the foetal stomach content for diagnosis of brucellosis, but not on the stomach tissue. However, this study also suggests that the foetal stomach is not a good sample to be considered for diagnosis of *B. melitensis* infection. This is based on the success results from the direct bacterial culture of only 45%. In this study, foetal stomach exhibited pathological changes in the form of necrosis of the stomach cells leading to infiltration of inflammatory cells in the stomach wall with presence of necrotic materials in the lumen of the stomach.

Necrotizing placentitis, although not a pathognomonic lesion, has been regarded as the epitome of brucellosis. This lesion is often characterised by presence of bacterial colonies within the epithelium, connective tissues or between the villi, extensive necrosis with appearance of eosinophilic debris, expansion of the lamina propria due to oedema, oedematous villi and infiltration of inflammatory cells (Yazicioglu and Haziroglu 2000; SCAHAW 2001; Al-Tememy et al. 2013). All of these lesions were observed in this experiment with varying severity with the placental tissues from group 4 exhibiting the most severe histological changes. The presence of *B. melitensis* in the placental tissues were confirmed via bacteriological, molecular and IP methods. The IP staining revealed that *B. melitensis* was distributed primarily in the endometrial glands and occasionally in the inter-cotyledonary areas and some trophoblasts as early as 15 days post-infection. Contamination of foetal membrane could be a major source of infection to other livestock animals, humans and surrounding wildlife that are in close contact with the infected goats (Bamaiyi et al. 2017).

Several studies have shown that brucellosis produces lesions in the foetal kidneys and IHC has the ability to demonstrate the presence *Brucellae* in the kidneys (Cetín et al. 2004; Sözmen et al. 2010). Similarly, this study revealed that foetal kidneys developed mild to moderate glomerulonephritis with severe and extensive necrosis. However, bacteriological and molecular studies concluded that kidneys are less reliable in detecting brucellosis in comparison to other organs.

Abortion occurs when there is an expulsion of a non-viable, premature foetus usually of more than 15 days before the predicted date of delivery (Blood and Studdert 1988). Stillborn is often defined as the birth of usually a full-term dead foetus (Blood and Studdert 1988). In this study, all foetuses in group 4 were indeed in their last trimester of pregnancy and were either aborted or stillborn. Trophoblasts are essential for protection of the foetal implantation and to support of growth and thus, destruction of these cells can lead to abortion (Al-Tememy et al. 2013). Many reports indicate the role of trophoblast in brucellosis as the target site for replication. This is where the bacterium thrives in the presence of erythritol to replicate exponentially resulting in placentitis, thus abortion is triggered when supplemented with the influence of hormones, which actually mimics the process of parturition (Gorvel 2008; Xavier et al. 2010; Petersen et al. 2013). In addition, *B. melitensis* primarily grows in the extra-cotyledonary trophoblasts before disseminating to infect the placental (cotyledonary) trophoblasts (Al-Tememy et al. 2013; Petersen et al. 2013), which is also similar with the findings of the current study.

It is indeed worthwhile to note that the infection by the recently identified Schmallenberg virus appears similar to brucellosis (Claine et al. 2015). It was found that infection during a critical period of gestation can lead to abortion, stillbirth or the birth of severely malformed offspring (Wernike et al. 2015). The infectious SBV is capable of persisting in the foetal envelopes of ewes until the moment of birth and this persistence is of at least 100 days (Poskin et al. 2017), similar to the observation with brucellosis in this study.

## 5. Conclusion

Infection of pregnant does with *B. melitensis* leads to infected foetus that resulted in abortion or stillbirth due to necrotic and inflammatory lesions of the placentome. Severity of lesions and extent of antigenic distribution in the infected foetus increased with time of infection. In the infected foetus, the antigen was found mostly in stomach content as well as liver and spleen tissue, making these organs as the most suitable samples for bacterial isolation.
